# Assessing the burden of osteoarthritis in Latin America: a rapid evidence assessment

**DOI:** 10.1007/s10067-022-06063-9

**Published:** 2022-01-29

**Authors:** Daniel Ciampi de Andrade, Diego Saaibi, Nicolas Sarría, Nora Vainstein, Leslie Cano Ruiz, Rolando Espinosa

**Affiliations:** 1 LIM‑62, Pain Center, Faculdade de Medicina da Universidade de São Paulo, Hospital das Clínicas, São Paulo, Brazil; 2 Center for Neuroplasticity and Pain, Department of Health Science and Technology, Aalborg University, Aalborg, Denmark; 3 Universidad Autonoma de Bucaramanga, Bucaramanga, Colombia; 4 Instituto Oulton, Universidad Católica de Córdoba, Córdoba, Argentina; 5 Pfizer, Buenos Aires, Argentina; 6 Rheumatology Department, National Institute of Rehabilitation, Av. México-Xochimilco # 289, Mexico City, 14389 Mexico

**Keywords:** Burden of disease, Latin America, Osteoarthritis, Quality of life

## Abstract

This rapid evidence assessment (REA) was conducted to explore the burden of weight-bearing joint osteoarthritis in the developing countries of Latin America. REA methodology used a standardized search strategy to identify observational studies published from 2010 to 23 April 2020 that reported outcomes pertaining to the epidemiology and humanistic or economic burden of weight-bearing osteoarthritis. Relevant data from each included study were used to populate bespoke data extraction tables and qualitatively analyzed. Thirteen publications were identified that reported on knee and hip osteoarthritis in the Latin American region. Overall prevalence of physician-diagnosed symptomatic knee osteoarthritis in adults ranged from 1.55% in Peru to 7.4% in Ecuador. Total prevalence of grade ≥ 2 radiographic knee osteoarthritis was 22% among those ≥ 39 years of age in Brazil and 25.5% among those ≥ 40 years of age in Mexico. The prevalence of symptomatic/radiographic knee osteoarthritis was 7.1% in people ≥ 18 years of age in Mexico and 17.6% among those ≥ 40 years of age. Prevalence of hip osteoarthritis was similar to or slightly lower than knee osteoarthritis. The limited data available indicates weight-bearing osteoarthritis negatively affects quality of life and that the economic burden may vary between countries with different healthcare systems. The limited evidence found in the published literature suggests the burden of osteoarthritis in Latin America is substantial. Our analysis identified several evidence gaps, particularly for health-related quality of life and socioeconomic outcomes. Further research is of particular importance in areas where government-subsidized healthcare and resources are scarce.

## Introduction

Chronic pain is defined as pain that occurs on most days for at least 3 months and it has been estimated to affect 18% of the general population in developing countries [1]. A frequent cause of chronic pain is osteoarthritis (OA), a degenerative joint disease that commonly affects weight-bearing joints such as the knee and hip. The primary symptoms include joint pain, stiffness, and activity limitation that may lead to long-term disability and the need for joint replacement [2]. The pathophysiology of OA includes cartilage degradation, bone remodeling, osteophyte formation, and synovial inflammation, which leads to pain, stiffness, swelling, and loss of normal joint function [3].

Typically, the prevalence and incidence of radiographic and symptomatic OA increases with age [2, 4] and women have a higher incidence of OA than men, especially after menopause [2]. The Global Burden of Disease 2010 study specifically ranked hip and knee OA the 11th highest contributor to global disability and 38th highest in disability-adjusted life years [5]. Hip and knee OA contribute significantly to the overall burden of OA, predominantly because of the need for joint replacement [5]. Pain and reduced physical function both significantly impact the health-related quality of life (HRQoL) of patients with OA [2]. The annual US economic burden of OA has been estimated at approximately $45 billion [6].

There is currently little evidence regarding the burden of OA in developing countries. Therefore, a rapid evidence assessment (REA) was conducted to investigate the burden of radiological, clinical, or subjective OA of all severities in weight-bearing joints, including the epidemiology, impact on quality of life, and economic burden in three geographic regions: Asia, Africa and the Middle East, and Latin America. We present here the results of this assessment for Latin America.

## Materials and methods

Our REA methodology used a standardized approach to identify observational (non-interventional) studies reporting outcomes pertaining to the epidemiology and humanistic or economic burden of weight-bearing OA. The protocol was prospectively registered with PROSPERO, an international database of systematic reviews in health and social care, welfare, public health, education, crime, justice, and international development, at https://www.crd.york.ac.uk/prospero/display_record.php?RecordID=180225. Searches for publications from 2010 onwards were conducted on April 23, 2020, in MEDLINE® via Ovid®, Embase® via Ovid®, and PubMed. Hand-screening of reference lists for relevant reviews was performed to identify any publications that may not have emerged in the database searches. This standardized approach was informed by the Preferred Reporting Items for Systematic Reviews and Meta-Analyses protocol (PRISMA-P) guidelines [7]. Prespecified inclusion/exclusion criteria based on the PICOS (population, intervention, comparator, outcome, study type) framework were used to design the search strategies (Table 1).

**Table 1:** PICOS inclusion and exclusion criteria

Population	Adults ≥ 18 years of age with radiological (x-ray, CT, or MRI), clinical or subjective osteoarthritis in weight-bearing joints, including terms such as gonarthrosis and coxarthrosis, in Asia, AfME, and LatAm regions
Intervention/comparator	Any intervention, any comparator
Outcomes	• Epidemiology, including but not restricted to, incidence, prevalence, mortality, death rate, case-fatality ratio • Quality of life • Economic burden, including but not restricted to, direct and indirect medical costs, quality-adjusted life years, disability-adjusted life years
Study types	Observational, real-world evidence
Exclusion criteria	• Animal, pilot, pre-clinical, or clinical intervention studies • Reviews, meta-analyses, congress abstracts, case reports, notes, comments, editorials, letters, or opinions • Studies reporting < 100 subjects • Studies reporting incidence, prevalence, or risk factors of pre- or post-operative surgical complications • Studies reporting economic modeling (cost-effectiveness and budget impact) • Not published in English • Published before 2010

*AfME*, Africa and the Middle East; *CT*, computed tomography; *LatAm*, Latin America; *MRI*, magnetic resonance imaging; *PICOS*, population, intervention, comparator, outcome, study type

Initially, the titles and abstracts of publications identified in the searches were screened by one reviewer for eligibility according to the inclusion and exclusion criteria, with a second reviewer performing a quality check of 10% of the screened publications. This was followed by screening of the full text of publications selected in the initial step, performed by the first reviewer, and a quality check of 10% of the selected publications by the second reviewer. Relevant data from each included study were used to populate bespoke data extraction tables. All included studies were systematically assessed for risk of bias using the modified Newcastle–Ottawa Scale tool for observational cross-sectional studies, and economic studies were assessed using the Joanna Brigg’s Institute Analysis of Cost, Technology and Utilization Assessment and Review Instrument (ACTUARI) [8]. Those studies with the lowest risk of bias and most relevant study design were prioritized in the interpretation of findings.

OA-specific HRQoL tools included Western Ontario and McMaster Universities Osteoarthritis Index (WOMAC),^1^ a widely used proprietary set of standardized questionnaires used to evaluate the condition of patients with OA of the knee and hip. The WOMAC evaluates three dimensions: pain, stiffness, and physical function with 5, 2, and 17 questions, respectively; each subscale is summated to a maximum score of 20, 8, and 68, respectively. There is also a total index score or global score, which is usually calculated by summing the three subscales. Lower scores indicate better levels of symptoms or less physical disability [9]. Items can also be rated on a five-level Likert scale (no difficulty to extremely difficult) or using a 0–100 mm visual analog scale (VAS) or an 11-point numeric rating scale (from 0 to 10). The symptom-specific VAS for pain is a horizontal line 10 cm (100 mm) long anchored by two descriptors, usually “no pain” and “worst imaginable pain,” at opposite ends of the scale. Scores are from 0–100 or 0–10, with higher scores representing more pain.

Qualitative analysis of the data was performed. Studies were categorized primarily by geographic region (Asia, Africa and the Middle East, and Latin America), and then secondarily by outcomes reported (epidemiologic, quality of life, economic), joint location (hip, knee), diagnosis (symptomatic, radiographic, or symptomatic/radiographic [using x-ray imaging in combination with either clinical criteria or self-reported pain]), population source, and severity.

## Results

**Fig. 1:**
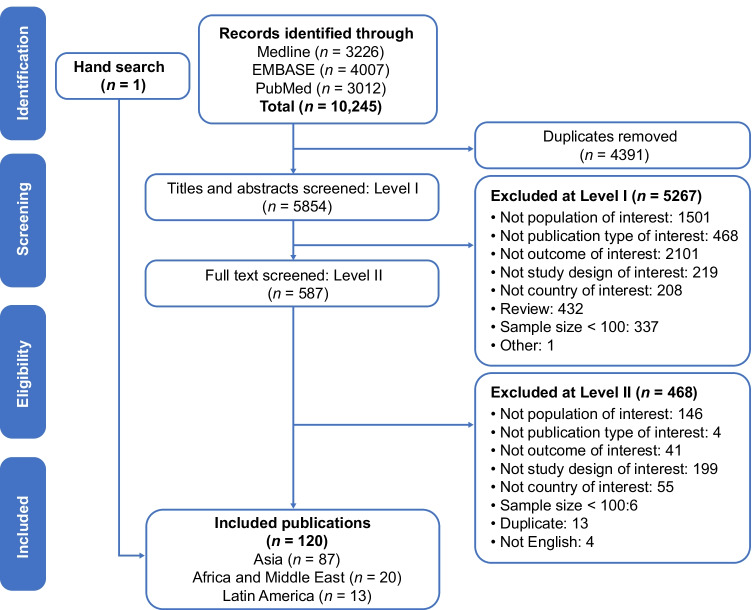
Search and screening flow chart

**Fig. 2:**
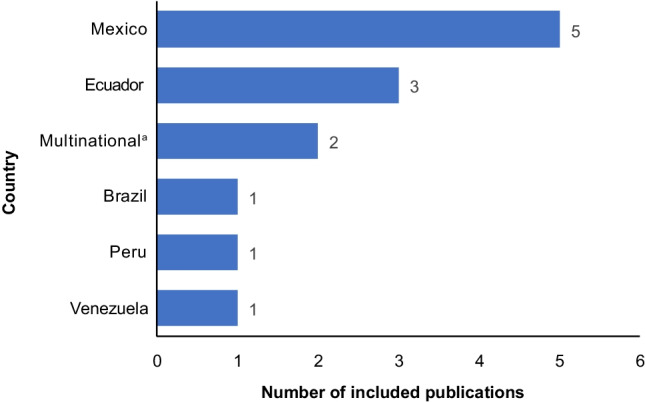
Countries included in 13 publications. ^a^Argentina, Bolivia, Chile, Colombia, Cuba, El Salvador, Guatemala, Mexico, Paraguay, Peru, Dominican Republic, Uruguay, Venezuela [20]; and Argentina, Brazil, and Mexico [10]

A total of 10,245 publications were identified by database searches. After removal of duplicates, 5854 were screened by titles and abstracts and 5267 were excluded for reasons shown in Fig. 1. Subsequently, 587 publications underwent full-text screening, 468 were excluded, and 1 additional publication identified through a hand search was added, resulting in 120 publications included from the three geographic areas (Fig. 1). Of these, 13 publications reported studies of populations from the Latin American region [10–22], with over 60% of studies from Mexico [11, 16–18, 22] and Ecuador [13–15] (Fig. 2). All of the 13 studies [10–22] reported knee OA and 5 (38%) studies [11, 17, 18, 20, 21] also reported hip OA. No other weight-bearing joints were reported.

The overall prevalence of physician-diagnosed symptomatic knee OA among men and women ≥ 18 years of age (Table 2) ranged from 1.55% in the Peruvian population [21] to 7.4% in Ecuador [14]. Among those ≥ 40 years of age in Mexico, the total prevalence of symptomatic knee OA was 19.6% [18]. Prevalence was significantly higher in women compared with men [13, 14, 18]. The total prevalence of grade ≥ 2 radiographic knee OA was 22% among those ≥ 39 years of age in a Brazilian study [19] and 25.5% among those ≥ 40 years of age in a Mexican study [18] (Table 3). In both studies, prevalence was highest for mild (grade 2) OA and decreased with increasing severity [18, 19]. In the Mexican study, prevalence was significantly higher in women compared with men (*p* = 0.02) and in the 40–49 years of age group compared with other age groups (*p* = 0.05) [18]. Furthermore, in the Mexican population, prevalence rates for symptomatic/radiographic knee OA were 7.1% in all adults ≥ 18 years of age in one study and 17.6% among those ≥ 40 years of age in another study [17, 18].

**Table 2:** Overall prevalence of symptomatic knee OA in 5 publications

First author, year/design	Registry, country	Population size, *n*			Population	Prevalence of knee OA, %		
		Total	Men	Women		Total	Men	Women
Granados, 2015 [12] cross-sectional	Venezuela	3973	1606	2367	Men and women ≥ 18 years of age	5.4	NR	NR
Guevara, 2019 [13] cross-sectional	Ecuador	2687	997	1690	Men and women ≥ 18 years of age	6.5	4.5	7.8*
Guevara-Pacheco, 2016 [14] cross-sectional	Ecuador	4877	1961	2916	Men and women ≥ 18 years of age	7.4	4.5	9.3**
Macias-Hernandez, 2018 [18] cross-sectional	Mexico	204	80	124	Men and women ≥ 40 years of age	19.6	12.5	24.2***
					Men and women 40–49 years of age	6.4	NR	NR
					Men and women 50–59 years of age	7.4	NR	NR
					Men and women 60–79 years of age	4.9	NR	NR
					Men and women > 80 years of age	0.98	NR	NR
Vega-Hinojosa, 2018 [21] cross-sectional	COPCORD, Peru	1095	481	614	Men and women ≥ 18 years of age	1.55	NR	NR

^*^p = 0.001 vs. men

^**^p < 0.01 vs. men

^***^p = 0.02 vs. men

*COPCORD*, Community Oriented Program for Control of Rheumatic Diseases; *NR*, not reported; *OA*, osteoarthritis

**Table 3:** Prevalence of radiographic knee OA in 2 publications

First author, year/design	Registry, country	Population size, *n*			Population	Severity (KL)	Prevalence of knee OA, %		
		Total	Men	Women			Total	Men	Women
Miguel, 2019 [19] cross-sectional	ELSA, Brazil	250	128	122	Men and women ≥ 39 years of age	Grade ≥ 2	22	NR	NR
						Grade 2	12.8	NR	NR
						Grade 3	4	NR	NR
						Grade 4	1.6	NR	NR
Macias-Hernandez, 2020 [18] cross-sectional	Mexico	204	80	124	Men and women ≥ 40 years of age	Grade ≥ 2	25.5	17.5	30.6*
					Men and women 40–49 years of age		11.8**	NR	NR
					Men and women 50–59 years of age		7.8**	NR	NR
					Men and women 60–79 years of age		4.9**	NR	NR
					Men and women > 80 years of age		0.98**	NR	NR
		204	80	124	Men and women ≥ 40 years of age	Grade 2	14.2	NR	NR
						Grade 3	7	NR	NR
						Grade 4	4	NR	NR

^*^p = 0.02 vs. males

^**^p = 0.05 between age groups

*ELSA*; Longitudinal Study of Adult Health; *KL*, Kellgren-Lawrence; *NR*, not reported; *OA*, osteoarthritis

The prevalence of symptomatic hip OA was reported in two studies [18, 21]. Among men and women ≥ 18 years of age in Peru, the prevalence was 0.37% [21]. Among those ≥ 40 years of age in Mexico, prevalence was 18.1% [18]. The prevalence of grade ≥ 2 radiographic hip OA in individuals ≥ 40 years of age in Mexico was 26.5% and was higher in women than men [18]. The prevalence of symptomatic/radiographic (grade ≥ 2) hip OA in this same population was 15.2% and was also higher in women than men [18].

**Fig. 3:**
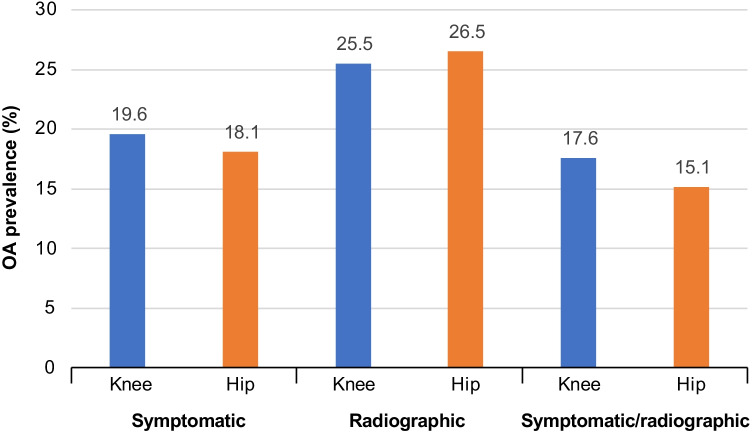
Comparison of prevalence of knee and hip OA by diagnostic methods in men and women ≥ 40 years of age in Mexico. *OA*, osteoarthritis

A comparison of the total prevalence of both hip and knee OA by three different diagnostic methods in a population ≥ 40 years of age in Mexico is shown in Fig. 3. The prevalence of grade ≥ 2 radiographic OA was higher than both symptomatic and combined symptomatic/radiographic OA for both knee and hip joints [18]. No studies were identified that reported incidence, survival/death rates, or all-cause mortality rates.

Patient-reported outcomes relating to HRQoL were described in two publications using the OA-specific WOMAC tool, the symptom-specific VAS pain scale, and two general HRQoL tools relating to disability (Health Assessment Questionnaire–Disability Index and Functioning Questionnaire). One study reported significant differences in pain severity and functional disability between populations with symptomatic knee OA from Mexico, Argentina, and Brazil, indicating less pain and generally better HRQoL despite a higher frequency of some functional limitation among patients in Mexico compared with Argentina and Brazil [10]. Another study from Ecuador reported significantly more physical disability in patients with symptomatic knee OA compared with the general population [15]. These results suggest that the quality of life of individuals with knee OA is negatively impacted and that the degree of OA burden may vary between countries.

Economic outcomes including direct costs and healthcare provision were reported in two studies, which found significant differences between patients with knee OA from Mexico, Argentina, and Brazil. Patients in Mexico were more likely to visit a primary care provider (37.6%, 21.4%, and 23.6% of patients in Mexico, Argentina, and Brazil, respectively) than a rheumatologist (16.1%, 41.0%, and 11.9%, respectively), possibly owing to disparities in health insurance coverage (95%, 29%, and 67% of patients in Mexico, Argentina, and Brazil, respectively, had no health insurance). No studies reported indirect costs. With the limited published information available, it was not possible to draw any conclusions about the economic burden of weight-bearing OA.

## Discussion

This analysis revealed a paucity of published literature regarding the burden of OA in Latin America. The occurrence of OA in weight-bearing joints was higher for the knee than the hip, in women compared with men, and in older populations compared with younger. The current analysis found a prevalence of symptomatic knee OA that ranged from 1.6 to 7.4% in adults ≥ 18 years of age [12–14, 21], and was 19.6% in an older population ≥ 40 years of age [18]. These findings are comparable to an estimated prevalence of symptomatic knee OA in the USA of 7.3% in men and women ≥ 25 years of age during 2011–2012, although the estimated prevalence of 5.7% in US Hispanics was slightly lower than the general population [23].

In our analysis, the prevalence of grade ≥ 2 radiographic knee OA was 22–25.5% among men and women ≥ 39–40 years of age [18, 19], and in one study was significantly higher in women than men [18]. A multinational systematic review and meta-analysis found a 24.5% prevalence of radiographic knee OA; however, that analysis found similar prevalence rates in women (27%) and men (26%) [24].

The total prevalence of symptomatic/radiographic knee OA was reported in three studies and ranged from 7 to nearly 18% [17–19]. As two of these studies involved Mexican populations, these differences are likely due to the age ranges; prevalence rates were 7% in men and women ≥ 18 years of age [17] and 17.6% in men and women aged ≥ 40 years [18]. However, the Brazilian study reported a prevalence of 9.6% for those ≥ 39 years of age; thus, differences may be due to ethnicity, patient demographics, OA etiology, or healthcare resource availability.

Prevalence rates varied widely due to heterogeneity between studies regarding diagnostic technique and population demographics (sex, age, general population vs OA patient population), highlighting the need for a standardized approach. Many studies were cross-sectional in design, with a risk of bias and possible misinterpretation. Several studies reported results from populations with self-reported diagnoses, which are subject to recall bias.

Evidence from patient-reported outcome tools showed a negative impact of knee OA on HRQoL, especially on pain and physical disability. Data from populations in Argentina, Brazil, and Mexico with symptomatic knee OA showed significant differences between countries [10]. Individuals in Mexico reported significantly more functional disability and worse WOMAC scores compared with people in Argentina and Brazil. Furthermore, the Mexican population reported significantly less pain on the VAS than Argentinian and Brazilian individuals. This study also reported on healthcare provision: 95% of Mexican subjects with knee OA had no health insurance compared with 29% of patients in Argentina and 67% in Brazil [10]. The authors commented that the healthcare system of the country of origin was more likely to be a predictor of pain and disability than ethnicity. Among individuals in Ecuador, 26% of those with symptomatic knee OA had physical disability, which was significantly greater than the 6.1% of the general population reporting disability [15]. The odds ratio for knee OA and physical disability was 5.08 (95% confidence interval: 3.6–7.0), suggesting that the subjects with knee OA are significantly impacted by disability compared with the healthy population [15]. Overall, our analysis showed that the humanistic burden was greatest in women and increased with age and OA severity.

There was limited evidence for economic burden in Latin America, with only two studies [10, 22] meeting the inclusion criteria for this analysis. Direct costs were most frequently reported and indicated a substantial financial burden, especially for imaging tests and knee OA requiring surgical intervention. No studies in our analysis reported indirect costs related to the economic burden of OA, such as loss of income due to absenteeism, reduced employment, early retirement, or informal caregiver costs. A study conducted in Canada reported that indirect costs accounted for 80% of the total economic burden to the individual with hip and knee OA [25]. Informal caregiving contributed an average of 40% to the total indirect costs, and employment-related costs were around 33% of the total economic burden [25].

There was a lack of evidence regarding socioeconomic burden, particularly for indirect costs relating to loss of earnings and informal care. Limited evidence suggested that the economic burden of disease varied according to the healthcare system within a country. Comparisons with published literature were complicated by lack of studies reporting direct costs in the same currency. Further research is needed to investigate the humanistic and economic burden on patients living in Latin American countries, and to understand the socioeconomic burden of disease on patients due to indirect costs.

An important limitation of the current analysis is that comparisons across studies were complicated owing to study heterogeneity, especially regarding diagnostic methods (symptomatic, radiographic, etc.) and population age and setting (general community, healthcare setting). Differences in diagnostic criteria (e.g., Kellgren-Lawrence cut-off grade) further complicated comparisons within a single diagnosis. It is also important to consider that the selection criteria for this review allowed only for the inclusion of studies that (1) specifically focused on studies with adult populations (≥ 18 years of age) with OA in weight-bearing joints only; (2) had ≥ 100 participants; and (3) were published in the English language. Any studies not meeting these criteria were excluded. Most of the included studies were cross-sectional in design, which has inherent limitations such as confounding factors. For example, age and gender may not be equally distributed between groups, leading to bias and misinterpretation. In addition, many studies investigated populations with self-reported diagnoses that are subject to recall bias. The population sampling method used (e.g., advertising for volunteers to investigate the impact of knee OA) was another factor contributing to the risk of bias.

In conclusion, the limited evidence from these analyses suggests that the burden of OA in weight-bearing joints is substantial in Latin America. Weight-bearing OA has a considerable negative impact on HRQoL. Limited economic evidence highlights an important burden that may be affected by the individual healthcare system within each country. This analysis identified several evidence gaps, particularly for HRQoL and socioeconomic outcomes. Future research should enable a better understanding of the burden of weight-bearing OA for patients and is of particular importance in areas where government-subsidized healthcare and resources are scarce.

## Footnotes

1 © 1996 Nicholas Bellamy. WOMAC® is a registered trademark of Nicholas Bellamy (CDN, EU, USA).
